# Use of Clinicians Who Focus on Nursing Home Care Among US Nursing Homes and Unplanned Rehospitalization

**DOI:** 10.1001/jamanetworkopen.2023.18265

**Published:** 2023-06-14

**Authors:** Seiyoun Kim, Kira L. Ryskina, Hye-Young Jung

**Affiliations:** 1 Division of General Internal Medicine, Department of Medicine, Perelman School of Medicine at the University of Pennsylvania, Philadelphia; 2 Division of Health Policy and Economics, Department of Population Health Sciences, Weill Cornell Medical College, New York, New York

## Abstract

**Importance:**

The number of physicians and advanced practitioners who focus their practice in nursing homes (NHs), often referred to as “SNFists” (ie, physicians, nurse practitioners, and physician assistants concentrating their practice in the nursing home or skilled nursing facility [SNF] setting) has increased dramatically. Little is known about the association of the NH medical care delivery models that use SNFists with the quality of postacute care.

**Objective:**

To quantify the association between NH use of SNFists and facility-level, unplanned 30-day rehospitalization rates for patients receiving postacute care.

**Design, Setting, and Participants:**

This cohort study used Medicare fee-for-service claims for all hospitalized beneficiaries discharged to 4482 NHs from January 1, 2012, through December 31, 2019. The study sample comprised NHs that did not have patients under the care of SNFists as of 2012. The treatment group included NHs that adopted at least 1 SNFist by the end of the study period. The control group included NHs that did not have patients under the care of a SNFist during the study period. SNFists were defined as generalist physicians and advanced practitioners with 80% or more of their Medicare Part B services delivered in NHs. Statistical analysis was conducted from January 2022 to April 2023.

**Exposure:**

Nursing home adoption of 1 or more SNFists.

**Main Outcomes and Measures:**

The main outcome was the NH 30-day unplanned rehospitalization rate. A facility-level analysis was conducted using an event study approach to estimate the association of an NH adopting 1 or more SNFists with its unplanned 30-day rehospitalization rate, adjusting for patient case mix, facility, and market characteristics. Changes in patient case mix were examined in secondary analyses.

**Results:**

In this study of 4482 NHs, adoption of SNFists increased from 13.5% of facilities (550 of 4063) in 2013 to 52.9% (1935 of 3656) in 2018. Adjusted rehospitalization rates were not statistically different after SNFist adoption compared with before, with an estimated mean treatment effect of 0.05 percentage points (95% CI, −0.43 to 0.53 percentage points; *P* = .84). The share of Medicare-covered patients increased by 0.60 percentage points (95% CI, 0.21-0.99 percentage points; *P* = .003) in the year of SNFist adoption and by 0.54 percentage points (95% CI, 0.12-0.95 percentage points; *P* = .01) 1 year after adoption compared with NHs that did not adopt SNFists. The number of postacute admissions increased by 13.6 (95% CI, 9.7-17.5; *P* < .001) after SNFist adoption, but there was no statistically significant change in the acuity index.

**Conclusions and Relevance:**

This cohort study suggests that NH adoption of SNFists was associated with an increase in the number of admissions for postacute care but was not associated with a change in rehospitalization rates. This may represent a strategy by NHs to maintain rehospitalization rates while increasing the volume of patients receiving postacute care, which typically results in higher profit margins.

## Key Points

**Question:**

What is the association between nursing home adoption of SNFists (ie, physicians, nurse practitioners, and physician assistants concentrating their practice in the nursing home or skilled nursing facility [SNF] setting) and facility-level, unplanned 30-day rehospitalization rates for patients receiving postacute care?

**Findings:**

In this national cohort study of 4482 US nursing homes using an event study approach, facility adoption of SNFists was not associated with statistically significant changes in rehospitalization rates.

**Meaning:**

This study suggests that SNFist adoption may represent a strategy by facilities to maintain rehospitalization rates while shifting the patient case mix toward patients receiving postacute care, which typically results in higher profit margins for nursing homes.

## Teaser

This cohort study quantifies the association between nursing home adoption of SNFists (ie, physicians, nurse practitioners, and physician assistants concentrating their practice in the nursing home or skilled nursing facility [SNF] setting) and facility-level, unplanned 30-day rehospitalization rates for patients receiving postacute care.

## Introduction

Nearly one-fifth of hospitalized Medicare beneficiaries are discharged to nursing homes (NHs), making them the most common facility destination and among the highest spending categories for postacute care.^1^ Deficits in postacute care outcomes in NHs—such as high rehospitalization rates—spurred Medicare to implement strategies, such as public reporting and value-based payments, to incentivize NHs to make investments in quality. At the same time, the number of physicians and advanced practitioners who practice almost exclusively in NHs, colloquially known as “SNFists” (ie, physicians, nurse practitioners, and physician assistants concentrating their practice in the nursing home or skilled nursing facility [SNF] setting), increased dramatically over the past decade.^2,3^ Little is known about the association of NH care models that include SNFists with the quality of postacute care in these facilities.

Evidence of the association of clinician specialization with care outcomes in other settings is mixed. For example, over the past 3 decades, most US hospitals have adopted hospitalists—physicians or advanced practitioners who specialize in inpatient care—to improve patient outcomes and reduce costs.^4,5,6,7^ Evaluations of hospitalist care models have reported reduced length of stay; lower mortality after hospitalization for pneumonia, heart failure, and orthopedic conditions; and lower costs for hospital care compared with the traditional inpatient care overseen by a rotating primary care attending physician.^5,8,9^ However, hospitalist care has also been associated with higher costs for postacute care.^6,10,11^ Studies of physician specialization in other areas have also found higher, potentially avoidable health care use associated with care provided by physicians who focus on a particular setting for patients with heart failure^12^ and those with dementia.^13^

As NHs face severe shortages of physicians and advanced practitioners who can provide on-site primary and urgent care to patients with medically complex conditions,^14^ the demand for SNFists is high. Thus, assessing the association of SNFist adoption with postacute care outcomes in NHs is timely and crucially important to inform policy and practice. In this study, our objective was to measure the association between NH adoption of SNFists and rates of unplanned 30-day rehospitalization. Thirty-day rehospitalizations are an important measure of care quality,^15^ not only for NHs but also for hospitals that face financial penalties from Medicare for poor performance in this area. Furthermore, rehospitalization from an NH frequently initiates a spiral of rehospitalization and deconditioning, leading to further loss of function and eventual long-term care placement.^16^ Thus, rehospitalization is an outcome of importance to patients, caregivers, facilities, and policy makers.

## Methods

In this cohort study, we conducted 4 analyses. First, we examined the prevalence of SNFist adoption among NHs from January 1, 2013, to December 31, 2018. Second, we compared the characteristics of NHs that did and those that did not adopt SNFists. Third, we used an event study analysis to derive adjusted estimates of the association between NH adoption of SNFists and 30-day rehospitalization rates. Fourth, we examined changes in NH patient case mix after SNFist adoption. The study was approved with a waiver of informed consent by the University of Pennsylvania Institutional Review Board and Centers for Medicare & Medicaid Services Privacy Board because the research using large administrative data sets could not be carried out practicably without a waiver of consent. This study followed the Strengthening the Reporting of Observational Studies in Epidemiology (STROBE) reporting guideline.

### Data Sources

Medicare fee-for-service claims for all hospitalized beneficiaries discharged to NHs from January 1, 2012, through December 31, 2019, were used to identify acute care hospital discharges followed by NH stays for postacute care. The Medicare Carrier File, which contains Part B professional claims, was linked with the Medicare Provider Analysis and Review file to identify NHs for which SNFists provided services. The Medicare Beneficiary Summary File was used to confirm patient enrollment in Medicare Parts A and B. The NH Minimum Data Set (MDS) was used to measure patient demographic characteristics and clinical conditions. SNFists were identified using the Medicare Data on Provider Practice and Specialty. The data were then aggregated to the NH level and merged with facility and market characteristics from Brown University’s LTCFocus,^17^ Provider of Services Current Files, and the Area Health Resource Files.

### Study Population

Our study sample was limited to NHs that did not have patients under the care of SNFists as of 2012, the first year of the study period. The treatment group included NHs that adopted at least 1 SNFist by the end of the study period. The control group included NHs that did not have patients under the care of a SNFist during the study period.

Of the 15 057 NHs with data available during the study period, we excluded facilities that did not have at least 1 year of data before and after the adoption of a SNFist (n = 6891). We also excluded NHs that switched back and forth between the treatment and control groups (n = 3675) because the purpose of the study was to observe the association of continued use of SNFists after adoption with the rehospitalization rate. Observations with missing values for any variables were excluded from the analyses (n = 9). The final study sample included 4482 unique NHs: 2064 that adopted SNFists and 2418 that did not adopt SNFists.

### Key Variables

The primary outcome was the NH 30-day unplanned rehospitalization rate based on the Centers for Medicare & Medicaid Services clinical quality measure definition for short-stay patients.^18^ Our secondary outcomes included patient case mix characteristics—the percentage of an NH’s residents covered by Medicare,^17^ the number of postacute care admissions, and the patient acuity index,^17^ which is calculated based on the number of residents needing assistance with activities of daily living (ADL) and receiving special treatment.

Our variable of interest was a binary indicator reflecting an NH’s adoption of at least 1 SNFist. Consistent with the literature, these clinicians were defined as physicians in generalist specialties (family practice, general practice, geriatric medicine, hospitalist, internal medicine, physical medicine, and rehabilitation) and advanced practitioners (nurse practitioners and physician assistants) with at least 80% of their services provided to patients in NHs, who had at least 100 service lines in total and 10 or more visits to the NH in a given year.^19^

We also included measures of patient, facility, and market characteristics. The first MDS admission assessment was used to obtain patient characteristics, which included age, sex, and self-reported race and ethnicity, as well as MDS-based clinical variables used by the Centers for Medicare & Medicaid Services for risk adjustment (eg, functional status, clinical conditions, clinical treatments, and clinical diagnoses).^18^ A complete list of measures used for risk adjustment in the analyses can be found in the eMethods in Supplement 1. Other patient characteristics included performance on the Cognitive Function Scale^20^ and the ADL score.^21^ Facility characteristics included the total number of beds, profit status, multifacility chain affiliation, presence of special care units, staffing levels, occupancy rate, and percentage of residents covered by Medicare and by Medicaid. We also measured the number of physicians in generalist specialties and advanced practitioners who provided care at the NH annually. County-level market variables included the Herfindahl-Hirschman index for market concentration, Value-Based Payment penetration rate, Medicare Advantage penetration rate, and median household income.

### Statistical Analysis

Statistical analysis was conducted from January 2022 to April 2023. We examined the prevalence of SNFist adoption among NHs from 2013 to 2018 in our sample by calculating the percentage of NHs that adopted a SNFist out of the total NHs included in our analysis for each year. Linear regression was used to test whether the yearly trend was statistically significant. In addition, we constructed a map of the percentage of NHs that adopted SNFists by state to examine geographic variation in the prevalence.

We conducted unadjusted comparisons of the characteristics of NHs that did and those did not adopt SNFists using 2-sample *t* tests for continuous measures and the Pearson χ^2^ test for categorical variables. For adjusted analyses, we used an event study approach that exploited the staggered adoption of SNFists by NHs to estimate the association between adoption of a SNFist and a facility’s rehospitalization rate. This method was chosen because the adoption year differed by NH. The estimated mean treatment effect for each period was based on doubly robust estimators^22^ incorporated within the difference-in-differences approach developed by Callaway and Sant’Anna.^23^ This approach accounts for variation in treatment timing and is robust to treatment effect heterogeneity. Estimates were adjusted for the patient, NH, and market characteristics already described. In addition, indicators for the NH and year were included in the regression model, and SEs were clustered at the facility level.

In secondary analyses, we examined changes in the percentage of the NH’s residents covered by Medicare,^17^ number of admissions for postacute care, and the mean acuity index^17^ before vs after SNFist adoption using the event study approach already described. As a sensitivity analysis, we reran the event study using a stricter definition of SNFists by requiring that at least 90% of services to have been provided in NHs instead of the 80% threshold used in the primary analyses. We also analyzed the performance separately for NHs that adopted a SNFist earlier and NHs that adopted a SNFist in later years of the study period. This was done by comparing NHs that adopted SNFists between 2013 and 2015 with those that did not adopt SNFists and by comparing NHs that adopted SNFists between 2016 and 2018 with facilities that did not adopt SNFists. We conducted additional subsample analyses of for-profit NHs and of nonprofit NHs, as well as for urban NHs and those in rural areas.

*P* values were from 2-sided tests, and results were deemed statistically significant at *P* < .05. All statistical analyses were conducted with Stata MP, version 17.1 (StataCorp LLC).

## Results

**Figure 1.**
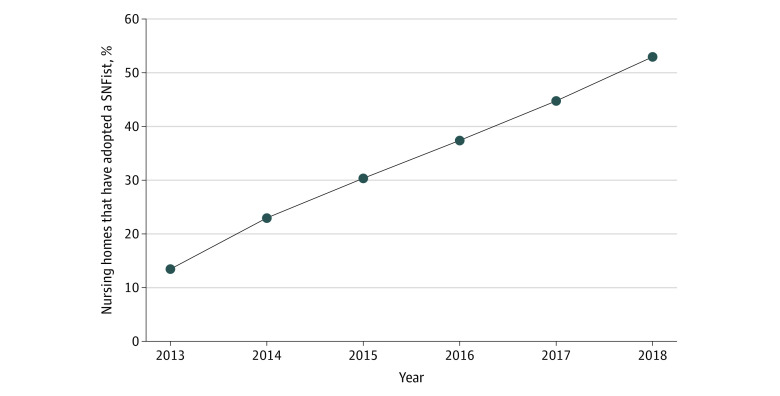
Prevalence of SNFist Adoption Among Nursing Homes This graph shows the prevalence of SNFist (ie, physicians, nurse practitioners, and physician assistants concentrating their practice in the nursing home or skilled nursing facility [SNF] setting) adoption among nursing homes over time.

**Figure 2.**
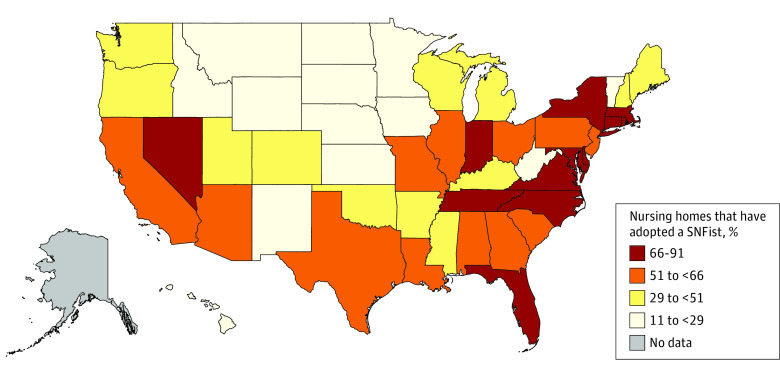
Nursing Home Adoption of SNFists by State This map shows the prevalence of SNFist (ie, physicians, nurse practitioners, and physician assistants concentrating their practice in the nursing home or skilled nursing facility [SNF] setting) adoption among nursing homes at the state level.

We observed increased adoption of SNFists over the study period. The percentage of NHs that adopted a SNFist among the total number of NHs in our sample increased from 13.5% (550 of 4063) in 2013 to 52.9% (1935 of 3656) in 2018 (*P* < .001) (Figure 1). Figure 2 shows the percentage of NHs that adopted SNFists by state. Nursing homes in Eastern states tended to have a higher prevalence of facilities that adopted SNFists, although additional states in the Midwest, South, and West Coast also had higher prevalences than other regions.

### Characteristics of NHs

Of the 4482 unique NHs in our sample, 2064 adopted a SNFist and 2418 did not adopt a SNFist during the study period. On average, patients in NHs that adopted a SNFist were younger (mean [SD] age, 77.7 [5.5] vs 79.3 [5.5] years; *P* < .001), more likely to include a higher percentage of individuals younger than 65 years (mean [SD], 14.5% [13.6%] vs 11.5% [12.8%]; *P* < .001), more racially diverse (mean [SD]: Black patients, 9.1% [15.1%]; White patients, 83.9% [20.3%]; and other race, 7.0% [13.5%] vs Black, 4.7% [11.8%]; White, 89.5% [17.4%]; and other race, 5.7% [12.7%]; *P* < .001), and more functionally impaired as indicated by higher ADL scores^21^ (mean [SD], 16.9 [2.3] vs 15.7 [2.7]; *P* < .001) compared with patients in NHs that never adopted a SNFist (Table).

**Table.** Baseline Characteristics of Nursing Homes That Did or Did Not Adopt SNFists

Characteristic	Never adopted SNFist (n = 2418)	Adopted SNFist (n = 2064)	*P* value^a^
**Patient characteristics**			
Age, mean (SD), y	79.3 (5.5)	77.7 (5.5)	<.001
Patient aged <65 y, mean (SD), %	11.5 (12.8)	14.5 (13.6)	<.001
Female, mean (SD), %	61.9 (11.7)	61.6 (10.4)	.32
Male, mean (SD), %	38.0 (11.7)	38.4 (10.4)	.32
Race and ethnicity			
Black, mean (SD), %	4.7 (11.8)	9.1 (15.1)	<.001
White, mean (SD), %	89.5 (17.4)	83.9 (20.3)	<.001
Other race or missing, mean (SD), %^b^	5.7 (12.7)	7.0 (13.5)	<.001
High CFS, mean (SD), %	6.1 (7.6)	6.2 (5.8)	.58
ADL at admission, mean (SD)	15.7 (2.7)	16.9 (2.3)	<.001
**Nursing home characteristics**			
Size, mean (SD), No. of beds	70.3 (42.0)	106.2 (53.4)	<.001
Profit status, No. (%)	1255 (51.9)	1475 (71.5)	<.001
Part of chain, No. (%)	1106 (45.7)	1164 (56.4)	<.001
Any special care unit, No. (%)	394 (16.3)	374 (18.1)	.11
CNA hours, mean (SD)	2.5 (1.2)	2.4 (0.8)	.006
LPN hours, mean (SD)	0.8 (0.6)	0.8 (0.4)	.43
RN hours, mean (SD)	0.7 (1.3)	0.5 (0.5)	<.001
Generalist physicians and advanced practitioners per resident, mean (SD)^c^	0.2 (0.6)	0.1 (0.2)	<.001
Occupancy rate, mean (SD), %	78.3 (17.2)	82.2 (14.4)	<.001
Medicaid patients, mean (SD), %	54.9 (25.6)	61.4 (22.3)	<.001
Medicare patients, mean (SD), %	15.5 (21.1)	15.3 (14.1)	.68
Star rating, No. (%)			
1	255 (10.5)	329 (15.9)	<.001
2	365 (15.1)	357 (17.3)	
3	476 (19.7)	464 (22.5)	
4	737 (30.5)	565 (27.4)	
5	524 (21.7)	309 (15.0)	
**Market characteristics**			
Market competition, mean (SD)	3326.3 (2070.9)	2596.9 (2033.5)	<.001
MA penetration, mean (SD)	18.0 (13.4)	22.9 (13.5)	<.001
VBP Program penetration, mean (SD)	0.02 (0.09)	0.01 (0.05)	<.001
Rural location, No. (%)	1645 (68.0)	708 (34.3)	<.001
Median income, mean (SD), $	46 049.3 (9911.6)	48 798.1 (12 288.9)	<.001

Abbreviations: ADL, activities of daily living; CFS, Cognitive Function Scale; CNA, certified nursing assistant; LPN, licensed practical nurse; MA, Medicare Advantage; RN, registered nurse; SNFist, physicians, nurse practitioners, and physician assistants concentrating their practice in the nursing home or skilled nursing facility setting; VBP, Value-Based Purchasing.

^a^ From the 2-sample *t* test for continuous variables and the Pearson χ^2^ test for categorical and binary variables.

^b^ Other race includes American Indian or Alaska Native, Asian, Hispanic, and Native Hawaiian or Other Pacific Islander. Multiracial people are also included in this category.

^c^ We include only physicians in generalist specialties (family practice, general practice, geriatric medicine, hospitalist, internal medicine, physical medicine, and rehabilitation). Number of residents was calculated by multiplying the total number of beds by the occupancy rate.

Facility characteristics differed between NHs that adopted SNFists and those that never adopted one. Nursing homes that adopted SNFists were larger on average (mean [SD], 106.2 [53.4] vs 70.3 [42.0] beds; *P* < .001), more likely to be for profit (1475 of 2064 [71.5%] vs 1255 of 2418 [51.9%]; *P* < .001), and more likely to be affiliated with a chain (1164 of 2064 [56.4%] vs 1106 of 2418 [45.7%]; *P* < .001) compared with NHs without a SNFist (Table). Staffing was generally lower in facilities that adopted a SNFist. Compared with never adopters, NHs adopting SNFists had lower certified nursing assistant hours per resident-day (mean [SD], 2.4 [0.8] vs 2.5 [1.2] hours; *P* = .006), similar licensed practical nurse hours per resident day (mean [SD], 0.8 [0.4] vs 0.8 [0.6] hours; *P* = .43), lower registered nurse hours per resident-day (mean [SD], 0.5 [0.5] vs 0.7 [1.3] hours; *P* < .001), and lower numbers of generalist physicians and advanced practitioners per resident (mean [SD], 0.1 [0.2] vs 0.2 [0.6]; *P* < .001). Nursing homes that adopted SNFists also had higher occupancy (mean [SD], 82.2% [14.4%] vs 78.3% [17.2%]; *P* < .001), had a higher percentage of patients covered by Medicaid (mean [SD], 61.4% [22.3%] vs 54.9% [25.6%]; *P* < .001), and were less likely to have a 5-star (highest) Care Compare NH rating (309 of 2064 [15.0%] vs 524 of 2418 [21.7%]; *P* < .001). Nursing homes that adopted a SNFist were in more concentrated markets (mean [SD] Herfindahl-Hirschman index, 2596.9 [2033.5] vs 3326.3 [2070.9]; *P* < .001), markets with a higher Medicare Advantage penetration rate (mean [SD], 22.9 [13.5] vs 18.0 [13.4]; *P* < .001), and markets with a lower value-based purchasing program penetration rate (mean [SD], 0.01 [0.05] vs 0.02 [0.09]; *P* < .001) compared with markets where NHs that never adopted a SNFist were located. Nursing homes adopting SNFists were in counties with a higher median income (mean [SD], $48 798.1 [$12 288.9] vs $46 049.3 [$9911.6]; *P* < .001) and were less likely to be located in rural counties (708 of 2064 [34.3%] vs 1645 of 2418 [68.0%]; *P* < .001) compared with NHs without SNFists.

We also conducted cross-temporal comparisons by examining the characteristics of NHs in our treatment and control groups both at the first year of observation and at the last year of observation. For most of the characteristics examined, the changes over time were in the same direction for both SNFist adopters and nonadopters (eTable 1 in Supplement 1). Some characteristics of NHs that were excluded from our analysis differed from those in our sample (eTable 2 in Supplement 1).

### Event Study Results

**Figure 3.**
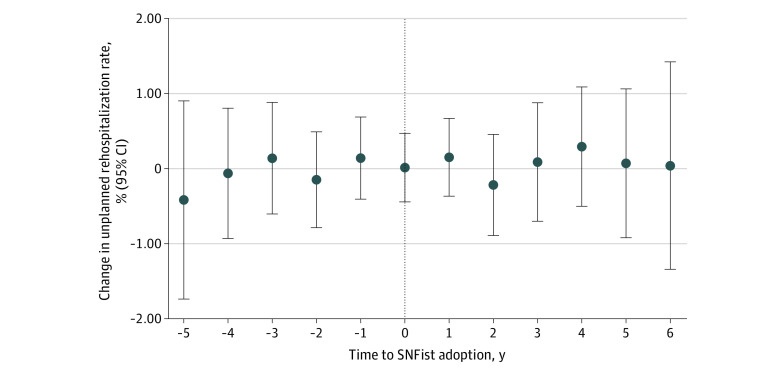
Event Study Estimates of Changes in Nursing Home Unplanned 30-Day Rehospitalization Rates Before and After SNFist Adoption Difference-in-differences estimators and their 95% CIs (indicated by error bars) are reported.

Adjusted estimates from our event study analysis did not indicate any statistically significant differences in rehospitalization rates between NHs that did and those that did not adopt SNFists (Figure 3; eTable 3 in Supplement 1). The estimated mean treatment effect was 0.05 percentage points (95% CI, −0.43 to 0.53 percentage points; *P* = .84).

**Figure 4.**
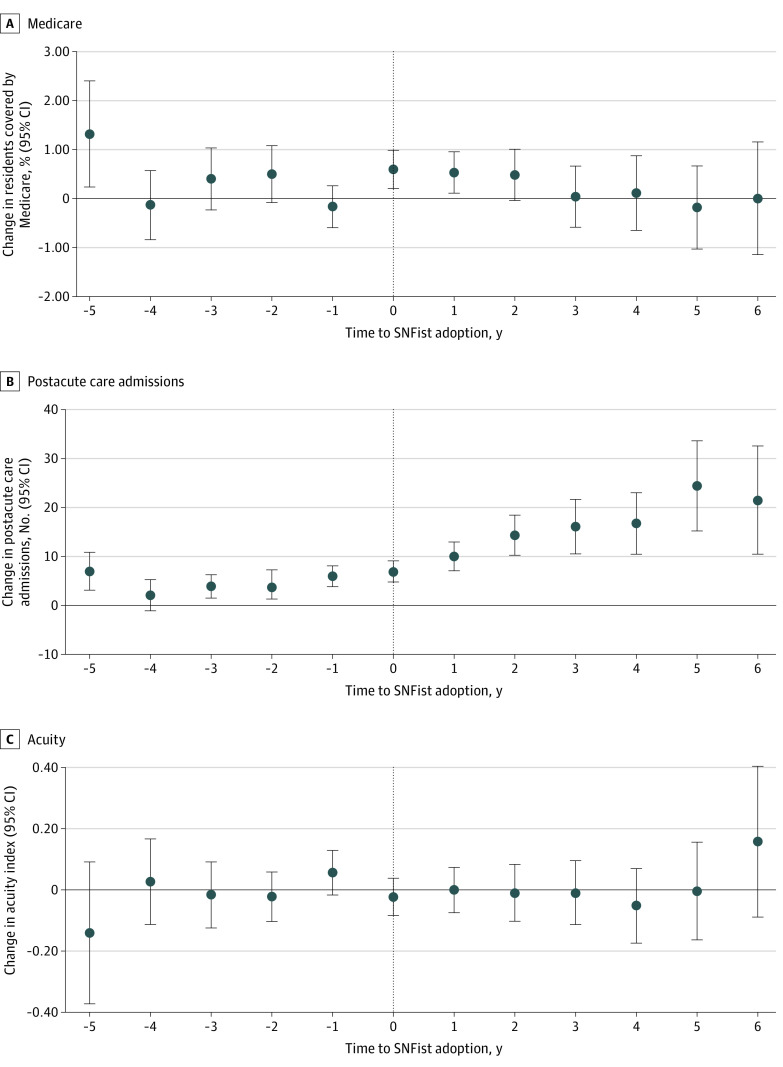
Event Study Estimates of Changes in Patient Mix A, Nursing home percentage of Medicare-covered patients. B, Number of new postacute admissions. C, Nursing home mean acuity index. Difference-in-differences estimators and their 95% CIs (indicated by error bars) are reported.

Figure 4 shows changes in the patient case mix before vs after SNFist adoption from our secondary analyses. The proportion of Medicare-covered residents increased by 0.60 percentage points (95% CI, 0.21-0.99 percentage points; *P* = .003) in the year of SNFist adoption and by 0.54 percentage points (95%, CI, 0.12-0.95 percentage points; *P* = .01) 1 year after adoption compared with NHs that did not adopt SNFists. We did not find statistically significant differences in other postadoption years. The number of postacute care admissions increased more quickly after the adoption period for NHs that adopted a SNFist compared with those that did not. The estimated mean treatment effect was 13.6 postacute care admissions (95% CI, 9.7-17.5 postacute care admissions; *P* < .001). With the mean number of postacute care admissions in our sample as the denominator (113.1), this represents a 12.0% relative increase in admissions. We did not find statistically significant changes in our secondary analysis examining NHs’ mean acuity index after SNFist adoption (Figure 4).

In our sensitivity analysis, in which a 90% threshold for the percentage of services provided in NHs was used to define SNFists, estimates did not indicate any statistically significant differences in rehospitalization rates associated with SNFist adoption (eFigure 1 in Supplement 1). The results of analyses stratified based on whether an NH adopted a SNFist earlier or later were consistent with the main analyses (eFigure 2 in Supplement 1). Similarly, we did not find any significant association in the subsample analyses of for-profit NHs and nonprofit NHs, as well as of facilities in urban and rural counties (eFigure 3 and eFigure 4 in Supplement 1).

## Discussion

In this national cohort study, we observed rapid adoption of SNFists by NHs. Although 30-day rehospitalization rates after SNFist adoption remained stable, NHs that adopted SNFists experienced an increase in the percentage of residents covered by Medicare and in the volume of patients admitted for postacute care. Adoption of SNFist-based care models by NHs may represent a strategy to maintain readmission rates while increasing the share of patients receiving postacute care in the facility.

These findings extend the literature on the role of clinician specialization in NH practice using doubly robust difference-in-differences estimators. Prior studies found that patients seen by clinicians who specialize in NH care were less likely to be rehospitalized in facilities with both types of clinicians^4^ and that increased use of NH specialists in a market was associated with lower use of antipsychotic medications and indwelling bladder catheters.^19^ However, both of these studies relied on cross-sectional study designs, which are vulnerable to confounding from unobserved differences in facility characteristics, including those of their patient populations, and between NHs with and those without SNFists. By using an event study, our approach mitigates the risk of unobserved confounding and provides estimates that are more suggestive of causal effects. Furthermore, the event study approach extends this body of literature by allowing us to examine heterogeneous associations of SNFist adoption with rehospitalization rates over time.

Although we did not observe an association between rehospitalization rates and SNFist adoption, we found that NHs that adopted SNFists experienced a shift in the patient case mix toward more postacute care (vs long-term care). Together, the absence of increases in rehospitalization rates suggests that SNFist adoption may reflect facility investments in care models better suited to managing patients receiving postacute care. This may be attractive to NHs because Medicare payments for postacute care are more profitable than Medicaid payments for long-term care.

Furthermore, hospitals are sensitive to increases in rehospitalization rates due to incentives created through programs such as the Hospital Readmissions Reduction Program, Medicare Shared Savings Program, and Bundled Payments for Care Improvement. Even small increases in rehospitalization rates may lead to hospitals and health systems reconsidering whether an NH should be part of their referral network.^24^ In addition, NHs incur financial penalties through the SNF VBP Program if they have high rehospitalization rates, currently the initiative’s only performance measure. The absence of a decrease in performance on this measure while increasing the volume of patients receiving postacute care after SNFist adoption may incentivize NHs to pursue this strategy for medical care delivery. Although the SNF VBP Program is set to end, it is expected to be replaced with a more multidimensional program that uses additional performance measures. For example, measures of patient satisfaction and communication with caregivers and families have been proposed for inclusion in future iterations of the NH VBP.^25^ Facilities that adopted SNFists may be making investments to improve these aspects of care in anticipation of these changes to the program.

### Limitations

Our study has several limitations. First, unobserved patient, NH, and market characteristics may have confounded the association between facility adoption of a SNFist and rehospitalization rates. However, we controlled for patient-, facility-, and market-level characteristics and included indicators for NHs in our analysis to account for any differences between facilities that do not vary over time. In addition, there were no statistically significant differences in pre-SNFist adoption trends in rehospitalization rates between NHs that did and those that did not adopt these clinicians. Second, we defined SNFists based on the relative volume of services provided in the NH setting and did not evaluate other characteristics of specialization, such as formal training, duration of practice in a given setting, or perceived expertise, among others. Experience and skill may affect outcomes but are difficult to measure.^26,27^

## Conclusion

This cohort study used a rigorous event study approach to isolate the association of SNFist adoption with NH rehospitalization rates, an important measure of postacute care quality that is salient to patients, caregivers, facilities, and policy makers. Facilities that adopted SNFists were able to shift the case mix to postacute care without an increase in rehospitalization rates, which may incentivize facilities to adopt models of care based on SNFists.

## Notes

Supplement 1.
eMethods.
**eTable 1.** Change in Nursing Homes Over Time **eTable 2.** Baseline Characteristics of Nursing Homes That Were Included and Excluded in the Analysis **eTable 3.** Adjusted Estimates From the Event Study **eFigure 1.** Event Study Estimates of Changes in Nursing Home Unplanned 30-Day Rehospitalization Rates When Defining SNFists Using a 90% Threshold for the Percentage of Their Evaluation and Management Services Provided in Nursing Homes **eFigure 2.** Event Study Estimates of Changes in Nursing Home Unplanned 30-Day Rehospitalization Rates for Analyses Stratified by Early Adopters and Later Adopters **eFigure 3.** Event Study Estimates of Changes in Nursing Home Unplanned 30-Day Rehospitalization Rates for Analyses Stratified by Nonprofit vs For-Profit Status **eFigure 4.** Event Study Estimates of Changes in Nursing Home Unplanned 30-Day Rehospitalization Rates Stratified by Rural vs Urban Location

Supplement 2.
Data Sharing Statement
