# Supplementary material for: Use of Clinicians Who Focus on Nursing Home Care Among US Nursing Homes and Unplanned Rehospitalization
Source: JAMA Netw Open. 2023 Jun 14;6(6):e2318265. doi: 10.1001/jamanetworkopen.2023.18265 (PMC10267770; doi:10.1001/jamanetworkopen.2023.18265)
Supplement: Supplement 1. — eMethods. eTable 1. Change in Nursing Homes Over Time eTable 2. Baseline Characteristics of Nursing Homes That Were Included and Excluded in the Analysis eTable 3. Adjusted Estimates From the Event Study eFigure 1. Event Study Estimates of Changes in Nursing Home Unplanned 30-Day Rehospitalization Rates When Defining SNFists Using a 90% Threshold for the Percentage of Their Evaluation and Management Services Provided in Nursing Homes eFigure 2. Event Study Estimates of Changes in Nursing Home Unplanned 30-Day Rehospitalization Rates for Analyses Stratified by Early Adopters and Later Adopters eFigure 3. Event Study Estimates of Changes in Nursing Home Unplanned 30-Day Rehospitalization Rates for Analyses Stratified by Nonprofit vs For-Profit Status eFigure 4. Event Study Estimates of Changes in Nursing Home Unplanned 30-Day Rehospitalization Rates Stratified by Rural vs Urban Location [file jamanetwopen-e2318265-s001.pdf]

Supplementary Online Content

Kim S, Ryskina KL, Jung HY. Use of clinicians who focus on nursing home care among US nursing homes and unplanned rehospitalization. *JAMA Netw Open.* 2023;6(6):e2318265. doi:10.1001/jamanetworkopen.2023.18265

eMethods.
eTable 1. Change in Nursing Homes Over Time
eTable 2. Baseline Characteristics of Nursing Homes That Were Included and Excluded in the Analysis
eTable 3. Adjusted Estimates From the Event Study
eFigure 1. Event Study Estimates of Changes in Nursing Home Unplanned 30-Day Rehospitalization Rates When Defining SNFists Using a 90% Threshold for the Percentage of Their Evaluation and Management Services Provided in Nursing Homes
eFigure 2. Event Study Estimates of Changes in Nursing Home Unplanned 30-Day Rehospitalization Rates for Analyses Stratified by Early Adopters and Later Adopters
eFigure 3. Event Study Estimates of Changes in Nursing Home Unplanned 30-Day Rehospitalization Rates for Analyses Stratified by Nonprofit vs For-Profit Status
eFigure 4. Event Study Estimates of Changes in Nursing Home Unplanned 30-Day Rehospitalization Rates Stratified by Rural vs Urban Location

This supplementary material has been provided by the authors to give readers additional information about their work.

© 2023 Kim S et al. *JAMA Network Open.*

eMethods.

MDS Risk Adjustment Variables for Unplanned Readmission

1) Functional Status

Acute change in mental status (C1600)
Rejected care for past four to seven days (E0800)
Wandering once or more in the past week (E0900)
Walks in room independently or with supervision or limited assistance (G0110C1)
Walks in corridor independently or with supervision or limited assistance (G0110D1)
Wanders and walks in room or corridor independently or with supervision or limited assistance (E0900, G0110C1 and G0110D1)
Two-person support needed with one or more ADLs (G0110A2 – G0110J2)
Coughing or choking during meals or when swallowing medications (K0100C)

2) Clinical Conditions

Shortness of breath with exertion (J1100A)
Shortness of breath when sitting at rest (J1100B)
End-stage prognosis (J1400)
Internal bleeding (J1550D)
Venous/Arterial ulcer present (M1030)
Surgical wound (M1040E)

3) Clinical Treatments

Ostomy care (H0100C)
Parenteral/IV feeding (K0510A2)
Feeding tube (K0510B2)
Antibiotic received (N0410F)
Chemotherapy for cancer (O0100A1 or O0100A2)
Radiation for cancer (O0100B1 or O0100B2)
Oxygen therapy (O0100C1 or O0100C2)
Ventilator or respirator (O0100F2)
IV medications (O0100H1 or O0100H2)
Transfusion (O0100I2)
Respiratory therapy (O0400D2)

4) Clinical diagnoses

Cancer (I0100)
Anemia (I0200)
Ulcerative Colitis/Crohn's disease/inflammatory bowel disease (I1300)
Viral hepatitis (I2400)
Alzheimer's disease (I4200)
Non-Alzheimer's dementia (I4800)
Seizure disorder or epilepsy (I5400)

5) Other

Returned to the nursing home following hospitalization (A1700 and A1800)
First assessment was for significant change in status (A0310A)

© 2023 Kim S et al. *JAMA Network Open.*

eTable 1. Change in Nursing Homes Over Time

	Never Adopted SNFist			Adopted SNFist		
Variables	First year of observation	Last year of observation	P-value[k]	First year of observation	Last year of observation	P-value[k]
Patient characteristics						
Age, mean (SD)	79.26 (5.52)	78.71 (5.68)	<.001	77.69 (5.47)	76.58 (5.46)	<.001
Patient under 65 %, mean (SD)	11.46 (12.81)	12.21 (13.01)	<.001	14.48 (13.58)	15.89 (13.57)	<.001
Female %, mean (SD)	61.95 (11.72)	60.26 (11.63)	<.001	61.62 (10.36)	57.66 (10.00)	<.001
Male %, mean (SD)	38.03 (11.71)	39.74 (11.63)	<.001	38.35 (10.36)	42.34 (10.00)	<.001
Race						
Black %, mean (SD)	4.73 (11.79)	5.05 (12.14)	<.001	9.13 (15.07)	10.48 (15.60)	<.001
White %, mean (SD)	89.54 (17.36)	86.83 (18.91)	<.001	83.88 (20.26)	79.39 (21.55)	<.001
Other race[a] %, mean (SD)	5.72 (12.75)	8.12 (15.04)	<.001	6.99 (13.47)	10.13 (15.41)	<.001
High CFS[b] %, mean (SD)	6.07 (7.61)	4.78 (7.21)	<.001	6.19 (5.77)	4.18 (4.60)	<.001
ADL[c] at admission, mean (SD)	15.66 (2.73)	15.83 (2.50)	.009	16.86 (2.34)	16.75 (2.07)	.01
Nursing home characteristics						
Size, mean (SD)	70.33 (41.98)	68.75 (37.42)	<.001	106.24 (53.39)	105.16 (51.20)	.003
Profit status, N (%)	1,255 (51.90%)	1,210 (52.82%)	.53	1,475 (71.46%)	1,500 (72.67%)	.39
Part of chain, N (%)	1,106 (45.74%)	1,128 (49.24%)	.02	1,164 (56.40%)	1,233 (59.74%)	.03
Any special care unit, N (%)	394 (16.29%)	299 (13.05%)	.002	374 (18.12%)	330 (15.99%)	.07
I[d] Hours, mean (SD)	2.47 (1.16)	2.39 (1.11)	.006	2.38 (0.79)	2.29 (0.80)	<.001
LPN[e] Hours, mean (SD)	0.82 (0.63)	0.81 (0.65)	.59	0.83 (0.41)	0.86 (0.39)	.001
RN[f] Hours, mean (SD)	0.70 (1.26)	0.72 (1.13)	.006	0.45 (0.49)	0.46 (0.44)	.03
Physician[g] and AP[h] per resident, mean (SD)	0.22 (0.61)	0.20 (0.61)	.80	0.12 (0.24)	0.14 (0.19)	<.001

© 2023 Kim S et al. *JAMA Network Open.*

	Never Adopted SNFist			Adopted SNFist		
Variables	First year of observation	Last year of observation	P-value[k]	First year of observation	Last year of observation	P-value[k]
Occupancy rate, mean (SD)	78.32 (17.21)	75.26 (18.00)	<.001	82.17 (14.40)	80.18 (14.47)	<.001
% Medicaid, mean (SD)	54.85 (25.59)	55.38 (25.59)	.25	61.42 (22.33)	60.30 (22.82)	.001
% Medicare, mean (SD)	15.54 (21.13)	13.59 (19.31)	<.001	15.32 (14.11)	13.75 (12.88)	<.001
Five star rating, N (%)			<.001			<.001
1	255 (10.55%)	199 (8.67%)		329 (15.94%)	301 (14.58%)	
2	365 (15.10%)	373 (16.28%)		357 (17.30%)	414 (20.06%)	
3	476 (19.69%)	392 (17.11%)		464 (22.48%)	352 (17.05%)	
4	737 (30.48%)	560 (24.44%)		565 (27.37%)	465 (22.53%)	
5	524 (21.67%)	764 (33.35%)		309 (14.97%)	531 (25.73%)	
Market characteristics						
Market competition, mean (SD)	3,326.33 (2,070.88)	3,440.83 (2,150.94)	<.001	2,596.95 (2,033.55)	2,687.95 (2,110.72)	<.001
MA[i] penetration, mean (SD)	18.05 (13.41)	23.83 (14.61)	<.001	22.93 (13.50)	32.61 (13.15)	<.001
VBP[j] penetration, mean (SD)	0.02 (0.09)	0.33 (0.28)	<.001	0.01 (0.05)	0.36 (0.23)	<.001
Rural location, N (%)	1,645 (68.03%)	1,534 (66.96%)	.43	708 (34.30%)	664 (32.17%)	.15
Median income, mean (SD)	46,049.27 (9,911.60)	55,144.50 (12,908.94)	<.001	48,798.11 (12,288.88)	61,008.23 (16,434.77)	<.001

Note: a. Other race includes American Indian/Alaskan Native, Asian, Hispanic, and Native Hawaiian/Pacific Islander. Multiracial people are also included in this category b. CFS=Cognitive Function Scale, c. ADL=Activities of Daily Living, d. I=Certified Nursing Assistant, e. LPN=Licensed Practical Nurse, f. RN=Registered Nurse, g. We only include physicians in generalist specialties (family practice, general practice, geriatric medicine, hospitalist, internal medicine, physical medicine, and rehabilitation). Number of residents was calculated by multiplying the total number of beds by the occupancy rate h. AP=Advanced Practitioner, i. MA=Medicare Advantage, j. VBP=Value Based Payment, k. P-values are from two sample t-test for continuous variables and Person's chi-squared test for categorical and binary variables.

For most of the variables examined, the changes over time were in the same direction for both SNFist-adopters and non-adopters. However, some noticeable differences were observed between the two groups in the mean ADL score, special care unit presence, LPN and RN hours, physician and AP per resident, and percentage of Medicaid covered patients. NHs that never adopted a SNFist experienced a statistically significant increase in mean ADL score (from

15.7 to 15.8; p=.009), decrease in special care unit presence (from 16.3% to 13.1%; p=.002), and increase in RN hours (from 0.70 to 0.72; p=.006), while no statistically significant change was observed for adopters. SNFist adopters had a statistically significant increase in LPN hours (from 0.8 to 0.9; p=.001), increase in physician and AP per resident (from 0.12 to 0.14; p<0.001), and decrease in Medicaid covered patients (from 61.4% to 60.3%; p<0.001) over time, while no statistically significant difference was observed for never adopters.

© 2023 Kim S et al. *JAMA Network Open.*

eTable 2. Baseline Characteristics of Nursing Homes That Were Included and Excluded in the Analysis

Variables	Included	Excluded	P-value[k]
Unique nursing homes, N	4,482	10,566	
Patient characteristics			
Age, mean (SD)	78.54 (5.55)	77.06 (5.62)	<.001
Patient under 65 %, mean (SD)	12.85 (13.25)	16.00 (14.31)	<.001
Female %, mean (SD)	61.80 (11.11)	60.78 (10.47)	<.001
Male %, mean (SD)	38.18 (11.10)	39.20 (10.46)	<.001
Race			
Black %, mean (SD)	6.76 (13.57)	11.68 (17.68)	<.001
White %, mean (SD)	86.94 (18.96)	79.26 (23.34)	<.001
Other race[a] or missing %, mean (SD)	6.30 (13.10)	9.06 (15.53)	<.001
High CFS[b] %, mean (SD)	6.13 (6.82)	6.16 (6.16)	.75
ADL[c] at admission, mean (SD)	16.21 (2.63)	17.04 (2.32)	<.001
Nursing home characteristics			
Size, mean (SD)	86.87 (50.82)	120.08 (64.82)	<.001
Profit status, N (%)	2,730 (60.91%)	7,904 (74.81%)	<.001
Part of chain, N (%)	2,270 (50.65%)	6,156 (58.26%)	<.001
Any special care unit, N (%)	768 (17.14%)	1,954 (18.49%)	.05
CNA[d] Hours, mean (SD)	2.43 (1.01)	2.37 (0.83)	<.001
LPN[e] Hours, mean (SD)	0.82 (0.54)	0.85 (0.50)	.001
RN[f] Hours, mean (SD)	0.58 (0.99)	0.48 (0.51)	<.001
Physician[g] and AP[h] per resident, mean (SD)	0.18 (0.48)	0.15 (0.28)	<.001
Occupancy rate, mean (SD)	80.10 (16.09)	83.49 (13.63)	<.001
% Medicaid, mean (SD)	57.88 (24.36)	60.03 (22.38)	<.001
% Medicare, mean (SD)	15.44 (18.23)	16.80 (14.71)	<.001
Five star rating, N (%)			<.001
1	584 (13.03%)	1,751 (16.57%)	
2	722 (16.11%)	2,227 (21.08%)	
3	940 (20.97%)	2,237 (21.17%)	
4	1,302 (29.05%)	2,718 (25.72%)	
5	833 (18.59%)	1,407 (13.32%)	
Market characteristics			
Market competition, mean (SD)	2,990.45 (2,085.48)	2,088.17 (1,875.23)	<.001
MA[i] penetration, mean (SD)	20.30 (13.67)	24.62 (13.12)	<.001
VBP[j] penetration, mean (SD)	0.01 (0.08)	0.01 (0.06)	<.001
Rural location, N (%)	2,353 (52.50%)	2,140 (20.25%)	<.001
Median income, mean (SD)	47,315.13 (11,153.18)	51,977.69 (13,380.89)	<.001

Note: a. Other race includes American Indian/Alaskan Native, Asian, Hispanic, and Native Hawaiian/Pacific Islander. Multiracial people are also included in this category b. CFS=Cognitive Function Scale, c. ADL=Activities of Daily Living, d. CNA=Certified Nursing Assistant, e. LPN=Licensed Practical Nurse, f. RN=Registered Nurse, g. We only include physicians in generalist specialties (family practice, general practice, geriatric medicine, hospitalist,

internal medicine, physical medicine, and rehabilitation). Number of residents was calculated by multiplying the total number of beds by the occupancy rate h. AP=Advanced Practitioner, i. MA=Medicare Advantage, j. VBP=Value Based Payment, k. P-values are from two sample t-test for continuous variables and Person's chi-squared test for categorical and binary variables.

NHs that were excluded from the sample had a higher percentage of patients under the age of 65 on average (16.0% vs. 12.9% vs p<.001) and were more racially diverse (Black: 11.7%, White: 79.3%, and other race: 9.1% vs. Black: 6.8%, White: 86.9%, and other race: 6.3%, p<.001), larger on average (120.1 vs. 86.9 beds, p<.001), more likely to be for-profit (74.8% vs. 60.9% p<.001), more likely to be affiliated with a chain (58.3% vs. 50.7%, p<.001), in addition to having a lower percentage with CMS Five-Star ratings (13.3% vs. 18.6%, p<.001) compared to NHs in our sample. Excluded NHs were also in more concentrated markets (Herfindahl–Hirschman index 2,088.2 vs. 2,990.5, p<.001) and included a lower percentage in rural counties (20.3% vs. 52.5.0%, p<.001).

© 2023 Kim S et al. *JAMA Network Open.*

eTable 3. Adjusted Estimates From the Event Study

Years Pre/Post SNFist Adoption	Rehospitalization Rate
-5 years	-0.419<br>(0.675)
-4 years	-0.064<br>(0.443)
-3 years	0.139<br>(0.379)
-2 years	-0.149<br>(0.326)
-1 years	0.140<br>(0.279)
0 years	0.014<br>(0.233)
1 years	0.150<br>(0.264)
2 years	-0.219<br>(0.343)
3 years	0.087<br>(0.403)
4 years	0.293<br>(0.406)
5 years	0.070<br>(0.506)
6 years	0.040<br>(0.705)
Number of observations	31,149
Number of facilities	4,482

Note: Standard errors in parentheses are clustered at facility, *** p<0.01, ** p<0.05, * p<0.1. Control variables include patient, facility, and regional level characteristics.

© 2023 Kim S et al. *JAMA Network Open.*

eFigure 1. Event Study Estimates of Changes in Nursing Home Unplanned 30-Day Rehospitalization Rates When Defining SNFists Using a 90% Threshold for the Percentage of Their Evaluation and Management Services Provided in Nursing Homes

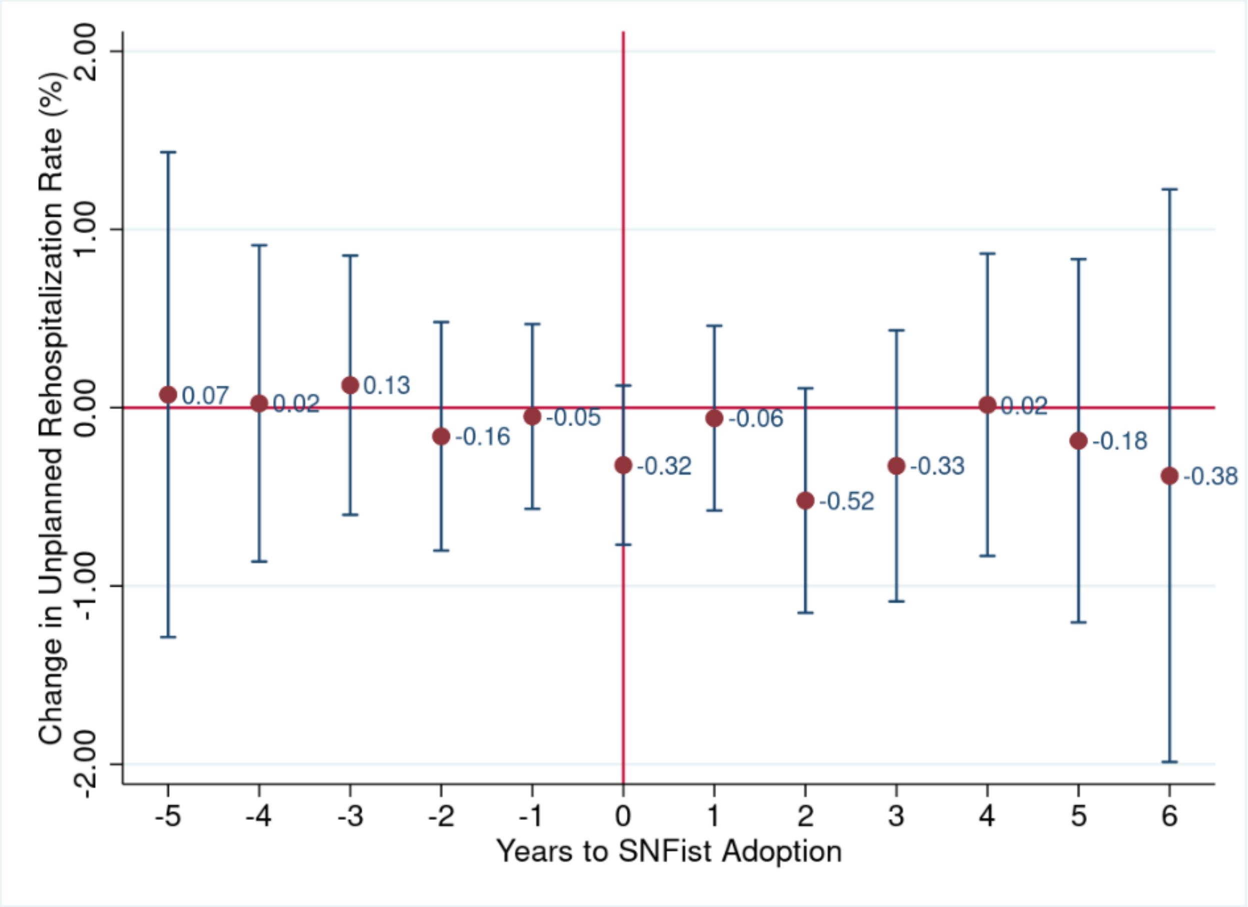

© 2023 Kim S et al. *JAMA Network Open*.

eFigure 2. Event Study Estimates of Changes in Nursing Home Unplanned 30-Day Rehospitalization Rates for Analyses Stratified by Early Adopters and Later Adopters

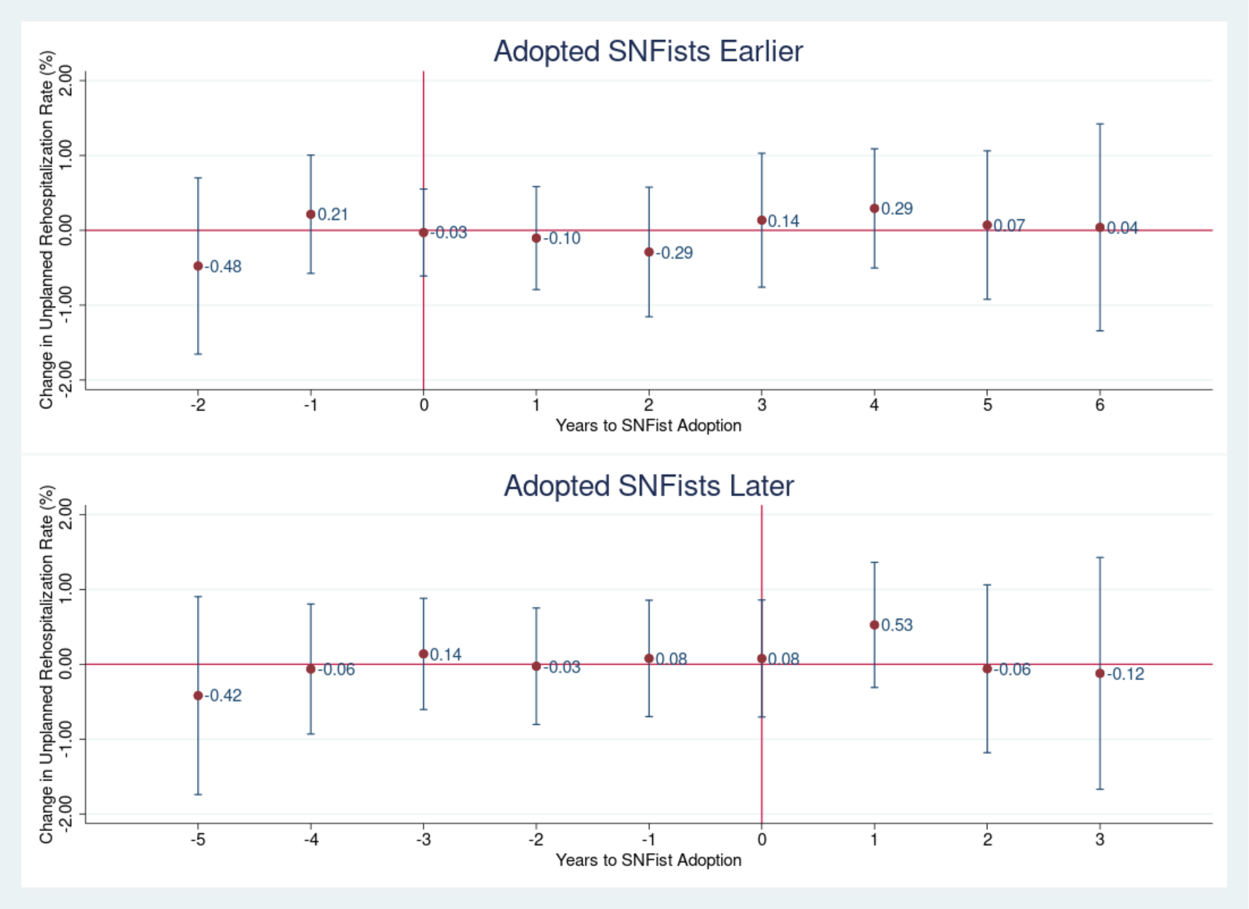

© 2023 Kim S et al. *JAMA Network Open.*

eFigure 3. Event Study Estimates of Changes in Nursing Home Unplanned 30-Day Rehospitalization Rates for Analyses Stratified by Nonprofit vs For-Profit Status

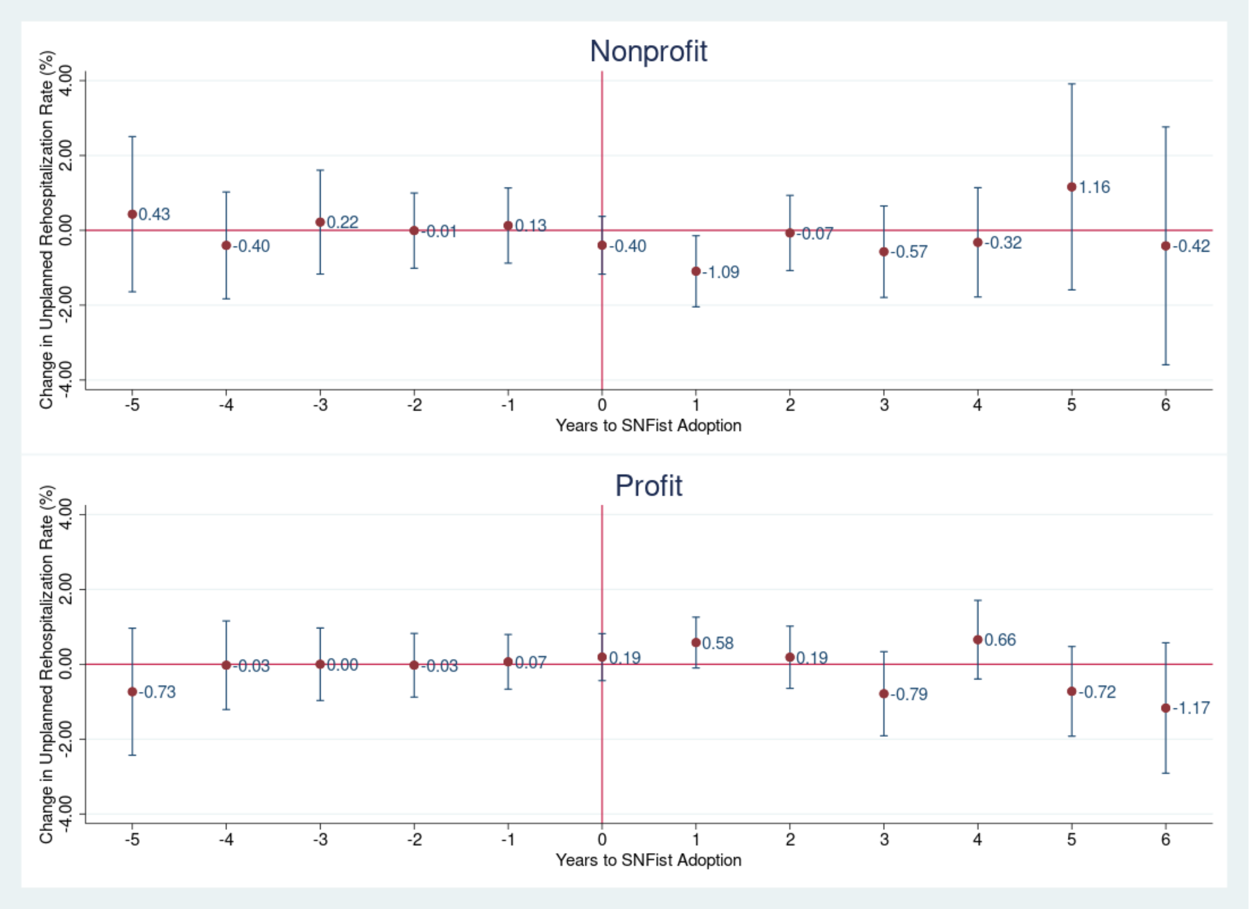

© 2023 Kim S et al. *JAMA Network Open.*

eFigure 4. Event Study Estimates of Changes in Nursing Home Unplanned 30-Day Rehospitalization Rates Stratified by Rural vs Urban Location

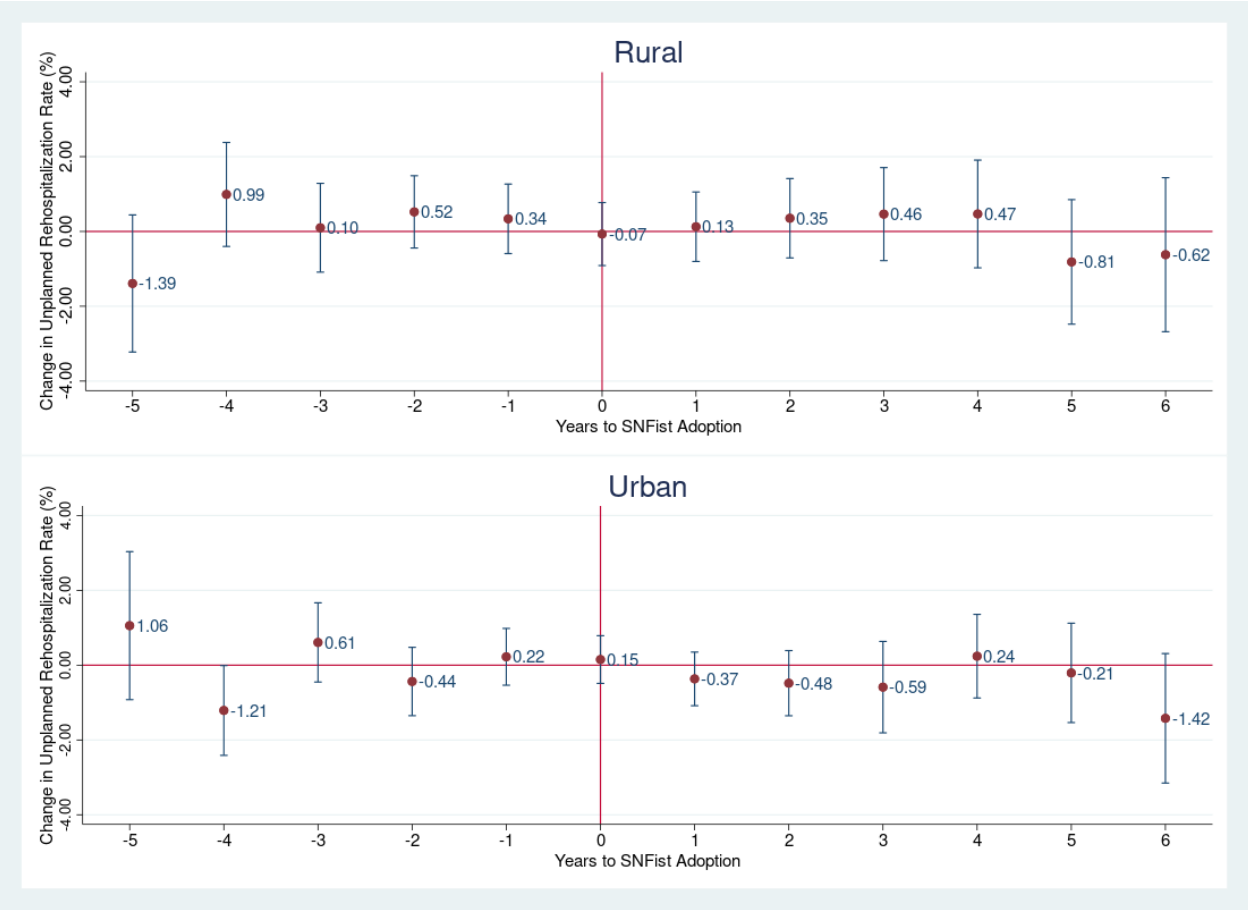

© 2023 Kim S et al. *JAMA Network Open.*
