# Supplementary material for: Use of Clinicians Who Focus on Nursing Home Care Among US Nursing Homes and Unplanned Rehospitalization
Source: JAMA Netw Open. 2023 Jun 14;6(6):e2318265. doi: 10.1001/jamanetworkopen.2023.18265 (PMC10267770; doi:10.1001/jamanetworkopen.2023.18265)
Supplement: Supplement 2. — Data Sharing Statement [file jamanetwopen-e2318265-s002.pdf]

Data Sharing Statement

Kim. Use of Clinicians Who Focus on Nursing Home Care Among US Nursing Homes and Unplanned Rehospitalization. *JAMA Netw Open*. Published June 14, 2023. doi:10.1001/jamanetworkopen.2023.18265

Data
Data available: No
